# Dichloridobis(4-methyl­benz­yl)(1,10-phenanthroline-κ^2^ *N*,*N*′)tin(IV)

**DOI:** 10.1107/S1600536810005672

**Published:** 2010-02-17

**Authors:** Thy Chun Keng, Kong Mun Lo, Seik Weng Ng

**Affiliations:** a Department of Chemistry, University of Malaya, 50603 Kuala Lumpur, Malaysia

## Abstract

The tin(IV) atom in the title compound, [Sn(C_8_H_9_)_2_Cl_2_(C_12_H_8_N_2_)], is chelated by the *N*-heterocycle and bonded to two C atoms and two chloride ions in an SnC_2_Cl_2_N_2_ octa­hedral coordination environment with the C atoms *trans* to each other. The dihedral angles between the heterocycle ring system and the benzene rings of the 4-methyl­benzyl groups are 21.20 (12) and 28.71 (11)°.

## Related literature

For the crystal structure of the 1,10-phenanthroline adduct with di(4-chloro­benz­yl)tin dichloride, see: Tan *et al.* (2009 ▶). For the direct synthesis of substituted dibenzyl­tin dichlorides, see: Sisido *et al.* (1961 ▶).
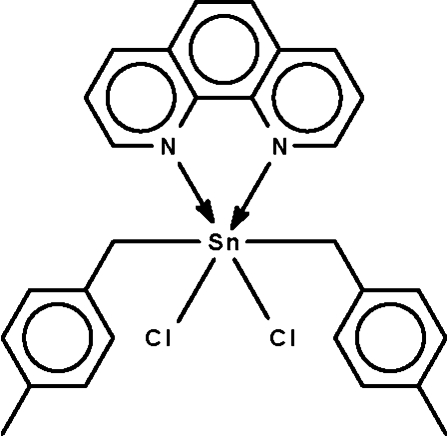


## Experimental

### 

#### Crystal data

[Sn(C_8_H_9_)_2_Cl_2_(C_12_H_8_N_2_)]
*M* *_r_* = 580.10
Monoclinic, 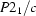

*a* = 15.2180 (2) Å
*b* = 10.4325 (1) Å
*c* = 17.7990 (2) Å
β = 115.0779 (5)°
*V* = 2559.42 (5) Å^3^
*Z* = 4
Mo *K*α radiation
μ = 1.23 mm^−1^
*T* = 293 K
0.40 × 0.30 × 0.10 mm

#### Data collection

Bruker SMART APEX CCD diffractometer
Absorption correction: multi-scan (*SADABS*; Sheldrick, 1996 ▶) *T*_min_ = 0.640, *T*_max_ = 0.887
23764 measured reflections
5882 independent reflections
5066 reflections with *I* > 2σ(*I*)
*R*_int_ = 0.023

#### Refinement

*R*[*F* ^2^ > 2σ(*F* ^2^)] = 0.025
*wR*(*F* ^2^) = 0.074
*S* = 1.05
5882 reflections
300 parameters
H-atom parameters constrained
Δρ_max_ = 0.43 e Å^−3^
Δρ_min_ = −0.68 e Å^−3^

### 

Data collection: *APEX2* (Bruker, 2009 ▶); cell refinement: *SAINT* (Bruker, 2009 ▶); data reduction: *SAINT*; program(s) used to solve structure: *SHELXS97* (Sheldrick, 2008 ▶); program(s) used to refine structure: *SHELXL97* (Sheldrick, 2008 ▶); molecular graphics: *X-SEED* (Barbour, 2001 ▶); software used to prepare material for publication: *publCIF* (Westrip, 2010 ▶).

## Supplementary Material

Crystal structure: contains datablocks global, I. DOI: 10.1107/S1600536810005672/hb5335sup1.cif

Structure factors: contains datablocks I. DOI: 10.1107/S1600536810005672/hb5335Isup2.hkl

Additional supplementary materials:  crystallographic information; 3D view; checkCIF report

## Figures and Tables

**Table 1:** Selected bond lengths (Å)

Sn1—C1	2.164 (2)
Sn1—C9	2.179 (2)
Sn1—N2	2.3595 (18)
Sn1—N1	2.3780 (18)
Sn1—Cl1	2.5256 (6)
Sn1—Cl2	2.5295 (7)

**Fig. 1.**
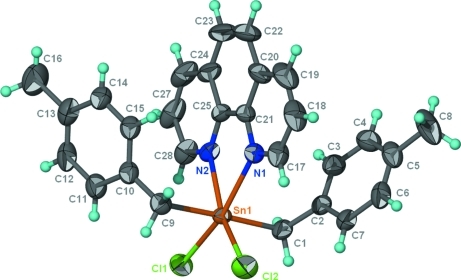
The molecular structure of (I) at the 50% probability level. Hydrogen atoms are drawn as spheres of arbitrary radius.

## supplementary crystallographic information

### Experimental

Di(*p*-methylbenzyl)tin dichloride was synthesized by the reaction of *p*-methylbenzyl chloride and metallic tin (Sisido *et al.*, 1961). The reactant (0.45 g, 1 mmol) and 1,10-phenanthroline (0.18 g, 1 mmol) were heated in chloroform (50 ml) for 1 hour. Faint-yellow, almost colourless, blocks of (I) separated from the cool solution after a day.

### Refinement

Hydrogen atoms were placed at calculated positions (C–H 0.93–0.96 Å) and were treated as riding on their parent atoms, with *U*(H) set to 1.2–1.5 times *U*_eq_(C).

### Crystal data

**Table:**

[Sn(C_8_H_9_)_2_Cl_2_(C_12_H_8_N_2_)]	*F*(000) = 1168
*M**_r_* = 580.10	*D*_x_ = 1.505 Mg m^−^^3^
Monoclinic, *P*2_1_/*c*	Mo *K*α radiation, λ = 0.71073 Å
Hall symbol: -P 2ybc	Cell parameters from 9858 reflections
*a* = 15.2180 (2) Å	θ = 2.3–28.3°
*b* = 10.4325 (1) Å	µ = 1.23 mm^−^^1^
*c* = 17.7990 (2) Å	*T* = 293 K
β = 115.0779 (5)°	Block, colourless
*V* = 2559.42 (5) Å^3^	0.40 × 0.30 × 0.10 mm
*Z* = 4	

### Data collection

**Table:**

Bruker SMART APEX CCD diffractometer	5882 independent reflections
Radiation source: fine-focus sealed tube	5066 reflections with *I* > 2σ(*I*)
graphite	*R*_int_ = 0.023
ω scans	θ_max_ = 27.5°, θ_min_ = 1.5°
Absorption correction: multi-scan (*SADABS*; Sheldrick, 1996)	*h* = −19→19
*T*_min_ = 0.640, *T*_max_ = 0.887	*k* = −13→13
23764 measured reflections	*l* = −23→22

### Refinement

**Table:**

Refinement on *F*^2^	Primary atom site location: structure-invariant direct methods
Least-squares matrix: full	Secondary atom site location: difference Fourier map
*R*[*F*^2^ > 2σ(*F*^2^)] = 0.025	Hydrogen site location: inferred from neighbouring sites
*wR*(*F*^2^) = 0.074	H-atom parameters constrained
*S* = 1.05	*w* = 1/[σ^2^(*F*_o_^2^) + (0.0413*P*)^2^ + 0.9608*P*] where *P* = (*F*_o_^2^ + 2*F*_c_^2^)/3
5882 reflections	(Δ/σ)_max_ = 0.001
300 parameters	Δρ_max_ = 0.43 e Å^−^^3^
0 restraints	Δρ_min_ = −0.68 e Å^−^^3^

### Fractional atomic coordinates and isotropic or equivalent isotropic displacement parameters (Å^2^)

**Table:**

	*x*	*y*	*z*	*U*_iso_*/*U*_eq_	
Sn1	0.258495 (11)	0.590677 (12)	0.707814 (8)	0.03991 (6)	
Cl1	0.28464 (5)	0.52229 (7)	0.85189 (4)	0.06375 (17)	
Cl2	0.22061 (7)	0.82753 (6)	0.70201 (5)	0.0763 (2)	
N1	0.24130 (14)	0.58938 (17)	0.56865 (11)	0.0451 (4)	
N2	0.28083 (14)	0.38087 (17)	0.66953 (12)	0.0450 (4)	
C1	0.41247 (18)	0.6286 (3)	0.75538 (14)	0.0521 (5)	
H1A	0.4292	0.6956	0.7969	0.063*	
H1B	0.4476	0.5519	0.7827	0.063*	
C2	0.44531 (17)	0.6680 (2)	0.69126 (13)	0.0490 (5)	
C3	0.4806 (2)	0.5793 (3)	0.65313 (16)	0.0568 (6)	
H3	0.4881	0.4943	0.6706	0.068*	
C4	0.5051 (2)	0.6148 (3)	0.58943 (18)	0.0673 (7)	
H4	0.5296	0.5534	0.5656	0.081*	
C5	0.49389 (19)	0.7397 (3)	0.56052 (16)	0.0674 (8)	
C6	0.4612 (2)	0.8295 (3)	0.60007 (18)	0.0650 (7)	
H6	0.4545	0.9145	0.5829	0.078*	
C7	0.43823 (19)	0.7948 (2)	0.66475 (16)	0.0561 (6)	
H7	0.4177	0.8573	0.6910	0.067*	
C8	0.5137 (3)	0.7780 (5)	0.4875 (2)	0.1062 (15)	
H8A	0.4542	0.8041	0.4425	0.159*	
H8B	0.5405	0.7064	0.4705	0.159*	
H8C	0.5590	0.8479	0.5031	0.159*	
C9	0.10328 (18)	0.5559 (2)	0.66245 (16)	0.0528 (5)	
H9A	0.0818	0.5933	0.7017	0.063*	
H9B	0.0706	0.6016	0.6105	0.063*	
C10	0.07041 (17)	0.4214 (2)	0.64869 (16)	0.0518 (5)	
C11	0.0640 (2)	0.3462 (3)	0.71101 (17)	0.0671 (7)	
H11	0.0804	0.3817	0.7632	0.081*	
C12	0.0337 (2)	0.2200 (3)	0.6968 (2)	0.0732 (8)	
H12	0.0306	0.1723	0.7398	0.088*	
C13	0.0078 (2)	0.1629 (3)	0.6200 (2)	0.0702 (8)	
C14	0.01504 (19)	0.2365 (3)	0.55829 (18)	0.0606 (6)	
H14	−0.0008	0.2005	0.5063	0.073*	
C15	0.04530 (17)	0.3623 (3)	0.57233 (15)	0.0532 (5)	
H15	0.0490	0.4092	0.5293	0.064*	
C16	−0.0291 (3)	0.0259 (4)	0.6033 (3)	0.1090 (13)	
H16A	−0.0768	0.0130	0.6246	0.164*	
H16B	0.0240	−0.0322	0.6301	0.164*	
H16C	−0.0579	0.0103	0.5446	0.164*	
C17	0.22739 (18)	0.6918 (3)	0.52146 (15)	0.0587 (6)	
H17	0.2259	0.7720	0.5437	0.070*	
C18	0.2148 (2)	0.6835 (4)	0.43852 (18)	0.0781 (9)	
H18	0.2076	0.7575	0.4073	0.094*	
C19	0.2132 (2)	0.5675 (4)	0.40508 (18)	0.0866 (11)	
H19	0.2032	0.5611	0.3499	0.104*	
C20	0.22645 (19)	0.4558 (4)	0.45285 (16)	0.0673 (8)	
C21	0.24283 (16)	0.4721 (2)	0.53616 (13)	0.0470 (5)	
C22	0.2244 (3)	0.3269 (5)	0.4227 (2)	0.0911 (12)	
H22	0.2131	0.3146	0.3676	0.109*	
C23	0.2380 (3)	0.2258 (4)	0.4710 (2)	0.0895 (12)	
H23	0.2338	0.1444	0.4484	0.107*	
C24	0.25889 (18)	0.2374 (3)	0.5567 (2)	0.0661 (8)	
C25	0.26192 (15)	0.3617 (2)	0.58928 (15)	0.0466 (5)	
C26	0.2746 (2)	0.1346 (3)	0.6110 (3)	0.0806 (10)	
H26	0.2721	0.0513	0.5916	0.097*	
C27	0.2933 (2)	0.1543 (2)	0.6910 (2)	0.0776 (9)	
H27	0.3037	0.0854	0.7269	0.093*	
C28	0.29680 (19)	0.2795 (2)	0.71911 (18)	0.0591 (6)	
H28	0.3109	0.2929	0.7747	0.071*	

### Atomic displacement parameters (Å^2^)

**Table:**

	*U*^11^	*U*^22^	*U*^33^	*U*^12^	*U*^13^	*U*^23^
Sn1	0.05738 (10)	0.03106 (8)	0.03553 (9)	−0.00481 (6)	0.02380 (7)	−0.00172 (5)
Cl1	0.0878 (4)	0.0687 (4)	0.0407 (3)	−0.0134 (3)	0.0330 (3)	0.0041 (3)
Cl2	0.1138 (6)	0.0339 (3)	0.1028 (6)	0.0027 (3)	0.0669 (5)	−0.0032 (3)
N1	0.0461 (10)	0.0525 (11)	0.0341 (9)	−0.0040 (8)	0.0145 (8)	0.0033 (7)
N2	0.0484 (10)	0.0345 (8)	0.0492 (10)	−0.0021 (7)	0.0178 (8)	−0.0019 (8)
C1	0.0595 (14)	0.0567 (13)	0.0350 (11)	−0.0159 (11)	0.0151 (10)	−0.0028 (10)
C2	0.0512 (12)	0.0536 (13)	0.0370 (10)	−0.0141 (10)	0.0138 (9)	−0.0036 (9)
C3	0.0604 (15)	0.0576 (15)	0.0492 (13)	−0.0031 (11)	0.0201 (11)	−0.0029 (10)
C4	0.0653 (16)	0.086 (2)	0.0521 (15)	−0.0013 (14)	0.0265 (13)	−0.0106 (14)
C5	0.0528 (14)	0.104 (2)	0.0446 (13)	−0.0111 (15)	0.0199 (11)	0.0084 (14)
C6	0.0607 (15)	0.0698 (17)	0.0642 (16)	−0.0142 (13)	0.0261 (13)	0.0119 (14)
C7	0.0618 (14)	0.0512 (14)	0.0580 (14)	−0.0150 (11)	0.0282 (12)	−0.0063 (11)
C8	0.085 (2)	0.178 (5)	0.068 (2)	0.005 (3)	0.0453 (18)	0.036 (2)
C9	0.0527 (13)	0.0535 (13)	0.0542 (13)	0.0024 (11)	0.0247 (11)	0.0005 (11)
C10	0.0438 (12)	0.0594 (14)	0.0518 (13)	−0.0039 (10)	0.0198 (10)	0.0023 (10)
C11	0.0654 (16)	0.084 (2)	0.0537 (14)	−0.0161 (15)	0.0273 (12)	0.0028 (14)
C12	0.0649 (16)	0.083 (2)	0.0739 (19)	−0.0168 (15)	0.0317 (14)	0.0190 (16)
C13	0.0601 (16)	0.0616 (17)	0.089 (2)	−0.0098 (13)	0.0315 (15)	0.0026 (15)
C14	0.0525 (13)	0.0616 (15)	0.0671 (16)	−0.0088 (12)	0.0247 (12)	−0.0071 (13)
C15	0.0467 (12)	0.0590 (14)	0.0508 (13)	−0.0032 (11)	0.0179 (10)	0.0056 (11)
C16	0.129 (3)	0.064 (2)	0.150 (4)	−0.023 (2)	0.075 (3)	−0.001 (2)
C17	0.0593 (14)	0.0635 (15)	0.0491 (13)	−0.0048 (12)	0.0188 (11)	0.0174 (11)
C18	0.0656 (17)	0.115 (3)	0.0493 (15)	−0.0040 (17)	0.0203 (13)	0.0327 (17)
C19	0.073 (2)	0.151 (4)	0.0376 (14)	−0.010 (2)	0.0253 (13)	0.0004 (18)
C20	0.0550 (14)	0.110 (2)	0.0410 (13)	−0.0119 (15)	0.0244 (11)	−0.0197 (14)
C21	0.0416 (11)	0.0619 (14)	0.0388 (11)	−0.0079 (10)	0.0182 (9)	−0.0116 (10)
C22	0.082 (2)	0.134 (3)	0.065 (2)	−0.024 (2)	0.0394 (17)	−0.056 (2)
C23	0.081 (2)	0.095 (3)	0.100 (3)	−0.016 (2)	0.046 (2)	−0.062 (2)
C24	0.0499 (13)	0.0592 (16)	0.091 (2)	−0.0072 (12)	0.0309 (13)	−0.0364 (15)
C25	0.0384 (10)	0.0470 (12)	0.0549 (13)	−0.0060 (9)	0.0202 (9)	−0.0162 (10)
C26	0.0644 (17)	0.0374 (13)	0.133 (3)	0.0004 (12)	0.0348 (19)	−0.0237 (17)
C27	0.0646 (17)	0.0312 (12)	0.120 (3)	0.0017 (11)	0.0232 (18)	0.0057 (15)
C28	0.0574 (14)	0.0413 (12)	0.0707 (16)	0.0045 (11)	0.0195 (12)	0.0080 (11)

### Geometric parameters (Å, °)

**Table:**

Sn1—C1	2.164 (2)	C11—C12	1.382 (4)
Sn1—C9	2.179 (2)	C11—H11	0.9300
Sn1—N2	2.3595 (18)	C12—C13	1.387 (4)
Sn1—N1	2.3780 (18)	C12—H12	0.9300
Sn1—Cl1	2.5256 (6)	C13—C14	1.383 (4)
Sn1—Cl2	2.5295 (7)	C13—C16	1.518 (5)
N1—C17	1.319 (3)	C14—C15	1.378 (4)
N1—C21	1.357 (3)	C14—H14	0.9300
N2—C28	1.332 (3)	C15—H15	0.9300
N2—C25	1.347 (3)	C16—H16A	0.9600
C1—C2	1.485 (3)	C16—H16B	0.9600
C1—H1A	0.9700	C16—H16C	0.9600
C1—H1B	0.9700	C17—C18	1.408 (4)
C2—C3	1.384 (4)	C17—H17	0.9300
C2—C7	1.393 (3)	C18—C19	1.345 (5)
C3—C4	1.386 (4)	C18—H18	0.9300
C3—H3	0.9300	C19—C20	1.406 (5)
C4—C5	1.384 (4)	C19—H19	0.9300
C4—H4	0.9300	C20—C21	1.406 (3)
C5—C6	1.385 (4)	C20—C22	1.443 (5)
C5—C8	1.507 (4)	C21—C25	1.440 (4)
C6—C7	1.385 (4)	C22—C23	1.320 (6)
C6—H6	0.9300	C22—H22	0.9300
C7—H7	0.9300	C23—C24	1.426 (5)
C8—H8A	0.9600	C23—H23	0.9300
C8—H8B	0.9600	C24—C26	1.394 (5)
C8—H8C	0.9600	C24—C25	1.413 (3)
C9—C10	1.475 (3)	C26—C27	1.345 (5)
C9—H9A	0.9700	C26—H26	0.9300
C9—H9B	0.9700	C27—C28	1.391 (4)
C10—C15	1.390 (4)	C27—H27	0.9300
C10—C11	1.395 (4)	C28—H28	0.9300
			
C1—Sn1—C9	178.47 (9)	C11—C10—C9	122.4 (2)
C1—Sn1—N2	91.02 (9)	C12—C11—C10	121.4 (3)
C9—Sn1—N2	90.27 (8)	C12—C11—H11	119.3
C1—Sn1—N1	91.80 (7)	C10—C11—H11	119.3
C9—Sn1—N1	89.41 (8)	C11—C12—C13	121.5 (3)
N2—Sn1—N1	70.10 (6)	C11—C12—H12	119.2
C1—Sn1—Cl1	88.62 (6)	C13—C12—H12	119.2
C9—Sn1—Cl1	90.50 (7)	C14—C13—C12	117.4 (3)
N2—Sn1—Cl1	92.93 (5)	C14—C13—C16	121.0 (3)
N1—Sn1—Cl1	163.03 (5)	C12—C13—C16	121.7 (3)
C1—Sn1—Cl2	91.59 (8)	C13—C14—C15	121.2 (3)
C9—Sn1—Cl2	87.42 (7)	C13—C14—H14	119.4
N2—Sn1—Cl2	162.30 (5)	C15—C14—H14	119.4
N1—Sn1—Cl2	92.32 (5)	C14—C15—C10	122.1 (2)
Cl1—Sn1—Cl2	104.63 (3)	C14—C15—H15	118.9
C17—N1—C21	119.1 (2)	C10—C15—H15	118.9
C17—N1—Sn1	125.14 (18)	C13—C16—H16A	109.5
C21—N1—Sn1	115.77 (14)	C13—C16—H16B	109.5
C28—N2—C25	118.8 (2)	H16A—C16—H16B	109.5
C28—N2—Sn1	123.61 (17)	C13—C16—H16C	109.5
C25—N2—Sn1	116.86 (15)	H16A—C16—H16C	109.5
C2—C1—Sn1	114.29 (15)	H16B—C16—H16C	109.5
C2—C1—H1A	108.7	N1—C17—C18	122.1 (3)
Sn1—C1—H1A	108.7	N1—C17—H17	118.9
C2—C1—H1B	108.7	C18—C17—H17	118.9
Sn1—C1—H1B	108.7	C19—C18—C17	119.2 (3)
H1A—C1—H1B	107.6	C19—C18—H18	120.4
C3—C2—C7	117.3 (2)	C17—C18—H18	120.4
C3—C2—C1	121.2 (2)	C18—C19—C20	120.5 (3)
C7—C2—C1	121.4 (2)	C18—C19—H19	119.7
C4—C3—C2	121.1 (3)	C20—C19—H19	119.7
C4—C3—H3	119.4	C21—C20—C19	116.9 (3)
C2—C3—H3	119.4	C21—C20—C22	118.1 (3)
C3—C4—C5	121.5 (3)	C19—C20—C22	125.0 (3)
C3—C4—H4	119.3	N1—C21—C20	122.1 (3)
C5—C4—H4	119.3	N1—C21—C25	118.45 (19)
C4—C5—C6	117.5 (2)	C20—C21—C25	119.4 (2)
C4—C5—C8	122.1 (3)	C23—C22—C20	122.0 (3)
C6—C5—C8	120.3 (3)	C23—C22—H22	119.0
C7—C6—C5	121.1 (3)	C20—C22—H22	119.0
C7—C6—H6	119.4	C22—C23—C24	122.1 (3)
C5—C6—H6	119.4	C22—C23—H23	119.0
C6—C7—C2	121.3 (3)	C24—C23—H23	119.0
C6—C7—H7	119.4	C26—C24—C25	117.1 (3)
C2—C7—H7	119.4	C26—C24—C23	124.8 (3)
C5—C8—H8A	109.5	C25—C24—C23	118.1 (3)
C5—C8—H8B	109.5	N2—C25—C24	121.8 (2)
H8A—C8—H8B	109.5	N2—C25—C21	118.1 (2)
C5—C8—H8C	109.5	C24—C25—C21	120.2 (2)
H8A—C8—H8C	109.5	C27—C26—C24	120.8 (3)
H8B—C8—H8C	109.5	C27—C26—H26	119.6
C10—C9—Sn1	117.26 (17)	C24—C26—H26	119.6
C10—C9—H9A	108.0	C26—C27—C28	118.9 (3)
Sn1—C9—H9A	108.0	C26—C27—H27	120.5
C10—C9—H9B	108.0	C28—C27—H27	120.5
Sn1—C9—H9B	108.0	N2—C28—C27	122.6 (3)
H9A—C9—H9B	107.2	N2—C28—H28	118.7
C15—C10—C11	116.4 (2)	C27—C28—H28	118.7
C15—C10—C9	121.1 (2)		
			
C1—Sn1—N1—C17	−85.6 (2)	C11—C12—C13—C14	1.1 (5)
C9—Sn1—N1—C17	93.4 (2)	C11—C12—C13—C16	−177.6 (3)
N2—Sn1—N1—C17	−176.1 (2)	C12—C13—C14—C15	−1.0 (4)
Cl1—Sn1—N1—C17	−176.82 (15)	C16—C13—C14—C15	177.7 (3)
Cl2—Sn1—N1—C17	6.02 (19)	C13—C14—C15—C10	0.3 (4)
C1—Sn1—N1—C21	96.79 (17)	C11—C10—C15—C14	0.4 (4)
C9—Sn1—N1—C21	−84.15 (17)	C9—C10—C15—C14	179.6 (2)
N2—Sn1—N1—C21	6.36 (15)	C21—N1—C17—C18	−0.5 (4)
Cl1—Sn1—N1—C21	5.6 (3)	Sn1—N1—C17—C18	−177.98 (19)
Cl2—Sn1—N1—C21	−171.54 (15)	N1—C17—C18—C19	2.5 (4)
C1—Sn1—N2—C28	90.9 (2)	C17—C18—C19—C20	−1.6 (5)
C9—Sn1—N2—C28	−88.3 (2)	C18—C19—C20—C21	−1.1 (5)
N1—Sn1—N2—C28	−177.6 (2)	C18—C19—C20—C22	178.9 (3)
Cl1—Sn1—N2—C28	2.21 (19)	C17—N1—C21—C20	−2.4 (3)
Cl2—Sn1—N2—C28	−170.65 (16)	Sn1—N1—C21—C20	175.29 (18)
C1—Sn1—N2—C25	−99.24 (16)	C17—N1—C21—C25	177.6 (2)
C9—Sn1—N2—C25	81.58 (17)	Sn1—N1—C21—C25	−4.7 (3)
N1—Sn1—N2—C25	−7.69 (15)	C19—C20—C21—N1	3.2 (4)
Cl1—Sn1—N2—C25	172.09 (15)	C22—C20—C21—N1	−176.7 (2)
Cl2—Sn1—N2—C25	−0.8 (3)	C19—C20—C21—C25	−176.8 (2)
N2—Sn1—C1—C2	83.16 (19)	C22—C20—C21—C25	3.3 (4)
N1—Sn1—C1—C2	13.03 (19)	C21—C20—C22—C23	−0.5 (5)
Cl1—Sn1—C1—C2	176.07 (19)	C19—C20—C22—C23	179.6 (3)
Cl2—Sn1—C1—C2	−79.34 (19)	C20—C22—C23—C24	−2.2 (6)
Sn1—C1—C2—C3	−94.0 (3)	C22—C23—C24—C26	−179.6 (3)
Sn1—C1—C2—C7	83.4 (2)	C22—C23—C24—C25	2.0 (5)
C7—C2—C3—C4	−1.9 (4)	C28—N2—C25—C24	−0.2 (3)
C1—C2—C3—C4	175.6 (2)	Sn1—N2—C25—C24	−170.55 (17)
C2—C3—C4—C5	−1.0 (4)	C28—N2—C25—C21	178.7 (2)
C3—C4—C5—C6	2.8 (4)	Sn1—N2—C25—C21	8.3 (3)
C3—C4—C5—C8	−175.7 (3)	C26—C24—C25—N2	1.2 (4)
C4—C5—C6—C7	−1.6 (4)	C23—C24—C25—N2	179.7 (2)
C8—C5—C6—C7	176.9 (3)	C26—C24—C25—C21	−177.7 (2)
C5—C6—C7—C2	−1.3 (4)	C23—C24—C25—C21	0.9 (3)
C3—C2—C7—C6	3.0 (4)	N1—C21—C25—N2	−2.4 (3)
C1—C2—C7—C6	−174.5 (2)	C20—C21—C25—N2	177.6 (2)
N2—Sn1—C9—C10	16.46 (19)	N1—C21—C25—C24	176.5 (2)
N1—Sn1—C9—C10	86.56 (19)	C20—C21—C25—C24	−3.5 (3)
Cl1—Sn1—C9—C10	−76.48 (19)	C25—C24—C26—C27	−1.0 (4)
Cl2—Sn1—C9—C10	178.91 (19)	C23—C24—C26—C27	−179.5 (3)
Sn1—C9—C10—C15	−90.4 (3)	C24—C26—C27—C28	0.0 (5)
Sn1—C9—C10—C11	88.7 (3)	C25—N2—C28—C27	−1.0 (4)
C15—C10—C11—C12	−0.3 (4)	Sn1—N2—C28—C27	168.7 (2)
C9—C10—C11—C12	−179.5 (3)	C26—C27—C28—N2	1.1 (5)
C10—C11—C12—C13	−0.4 (5)		
